# Effectiveness of Exercise Programs for Alleviation of Upper Body Pain in Patients with Spinal Cord Injury: A Systematic Review

**DOI:** 10.3390/jcm13113066

**Published:** 2024-05-23

**Authors:** Jiyoung Park, Jihyun Kim, Seon-Deok Eun, Dongheon Kang

**Affiliations:** 1 Department of Safety and Health, Wonkwang University, Iksan 54538, Republic of Korea; withji0@wku.ac.kr; 2 Department of Healthcare and Public Health Research, National Rehabilitation Center, Ministry of Health and Welfare, Seoul 01022, Republic of Korea; kjh0515@korea.kr

**Keywords:** spinal cord injury, upper body pain, shoulder pain, wheelchair, strength

## Abstract

**(1) Background:** Upper body pain, particularly in the limbs and shoulders, is a common symptom among patients with spinal cord injury (SCI) and wheelchair users. Despite the focus on resistance muscle training as a suitable intervention for SCI individuals, findings across different populations and conditions have been inconsistent. **(2) Methods:** We conducted a systematic review to elucidate the correlations among exercise interventions, muscle strength enhancement, and pain reduction. A comprehensive literature search was performed using the keywords “spinal cord injury,” “pain,” “exercise,” “disability,” “paraplegia,” and “tetraplegia” across the DBpia, EMBASE, PubMed, and Science Direct databases. **(3) Results:** From 191 identified articles, 13 studies (1 from Korea and 12 from other countries) were selected for analysis. The results indicate that exercise interventions are effective in reducing pain in patients with SCI, with a particular emphasis on alleviating shoulder pain. **(4) Conclusion:** Exercise is essential for pain reduction in patients with SCI, especially those experiencing shoulder pain. However, there is a notable lack of experimental research focusing primarily on pain. The development of appropriate measurement instruments is crucial for the prevention and relief of pain in this patient population.

## 1. Introduction

Spinal Cord Injury (SCI) is a severe medical condition resulting from damage to the spinal cord that affects motor, sensory, and autonomic functions. This introductory section aims to delineate the epidemiology of SCI, define and classify the associated pain types, identify the causes of pain specific to SCI patients, and discuss relevant pain scoring systems. SCI primarily results from traumatic events such as vehicular accidents, falls, sports injuries, or violent acts. The global incidence of SCI varies, but it is estimated that there are approximately 54 cases per million people per year, which emphasizes this condition’s significant public health burden [1]. The epidemiological data highlight the need for targeted preventive measures and improved healthcare strategies to manage the long-term outcomes of SCI. Pain in SCI can be broadly classified into nociceptive and neuropathic pain. Nociceptive pain is caused by damage to non-neural tissue and is characterized by aching or throbbing in muscles or joints. In contrast, neuropathic pain occurs directly due to spinal cord damage and is often described as sharp, intense, or burning [1,2]. Understanding these distinctions is crucial for accurate diagnosis and treatment. Pain in SCI patients can stem from various sources. Neuropathic pain can arise from the injury site itself or from dysfunctional nerve fibers. Nociceptive pain may be related to overuse of certain muscle groups or joints due to altered mobility patterns [2]. Secondary complications such as infections, pressure sores, and urinary tract infections can also contribute to pain in SCI patients.

Upper limb and shoulder pain are some of the most common musculoskeletal pains experienced by patients with spinal cord injuries (SCI) [1]. Wheelchairs are used for mobility and activities of daily living among patients with SCI with complete or incomplete lower limb functional impairments, and more than 50% of wheelchair users suffer from injuries and pain due to the overuse of their upper limbs [2]. The upper limbs bear repetitive strain during everyday wheelchair use, leading to shoulder joint damage and various pains and diseases [3]. According to Dalyan et al. [4], 76 out of 130 patients with SCI (58.5%) suffered from upper extremity pain, with 71% reporting shoulder pain, 53% with wrist pain, 43% with hand pain, and 35% with elbow pain, and many patients experience pain in more than one location. Shoulder damage and pain are the most characteristic symptoms for patients with SCI using manual wheelchairs, and these lead to disorders such as mechanical impingement syndrome or rotator cuff injury [5,6], as well as excessive use of other upper extremity muscles, such as the wrists. The continued use of strong force to maneuver the wheelchair, even with decreased mobility in the elbow and wrist due to fatigue following a shoulder injury, has a detrimental effect on wrist muscle function, leading to wrist pain and potentially causing carpal tunnel syndrome [7].

Therefore, for patients with SCI to use manual wheelchairs requiring upper body strength effectively, they must be free of musculoskeletal disorders or pain in the upper body. Moreover, pain prevention and alleviation are crucial, as prolonged use of upper body strength, function, and endurance for mobility increases the risk of pain [8]. Various exercise interventions have been developed and studied for their efficacy in improving upper-body muscle strength and pain relief in patients with SCI. Resistance training, as highlighted in a prior study [9], improves skeletal muscle strength and overall physical function for individuals seeking enhanced physical capabilities and has been consistently chosen as the intervention method in numerous studies focusing on patients with pain and SCI. A statistical analysis of muscle strength changes following an eight-week intervention via resistance and stabilization exercises in male patients with chronic neck pain revealed significant improvements in maximum muscle strength and increased maximum extension strength at varying cervical flexion angles, as well as significant neck pain relief in the resistance exercise group [10]. According to Kim and Song [11], resistance exercises using elastic bands in patients with SCI significantly improved isokinetic muscle strength during right internal and external rotation of the shoulder joint and right extension of the elbow joint, compared with the control group. Hicks et al. [12] also reported that patients with SCI attained improved muscle strength and reduced depression, stress, and pain by regularly engaging in physical activities.

Conversely, studies have also shown that using manual wheelchairs improved the physical fitness of patients with SCI [13] and that patients with SCI using wheelchairs had stronger shoulder function than patients with SCI with incomplete ambulation [14]. Furthermore, a study analyzing the correlations between physical activity levels and physical fitness parameters among patients with SCI [15] revealed almost no correlation between physical activity levels, types of activity, and upper limb muscle strength, indicating a low association level. These inconsistencies in study findings across study populations and conditions necessitate a systematic review of previous studies to clarify the correlations among (1) exercise interventions, (2) improvement of muscle strength, and (3) pain relief.

This study aimed to conduct a systematic review of studies conducted in Korea and abroad that have used changes in pain after exercise intervention in patients with SCI as the dependent variable and to investigate whether exercise interventions effectively alleviate upper body pain in patients with SCI. Based on the findings, we hypothesized that recommendations can be made for future studies and implications for alleviating upper body pain in patients with SCI.

## 2. Materials and Methods

### 2.1. Study Planning

Our systematic review followed the Preferred Reporting Items for Systematic Reviews and Meta-Analyses (PRISMA) guidelines. A statement was prepared in accordance with the PRISMA 2020 guidelines to ensure comprehensive reporting. The study procedure was as follows: (1) study planning, (2) literature search, (3) literature selection and categorization, (4) data analysis and results, and (5) discussion and conclusion. We employed the PICOS framework during the study planning phase to delineate the study objectives (Table 1). Specifically, P (Participants) comprised individuals with SCI experiencing upper body pain. I (Intervention) involved exercise, with C (Comparison) representing the control group in randomized controlled trials. O (Outcome) focused on changes in pain, and S (Study Design) indicated experimental studies. Notably, for P (Participants), individuals with lower back pain were intentionally excluded from the study. Using this PICOS strategy, we aimed to investigate improving musculoskeletal pain—the most common cause of upper body pain—through exercise, focusing on the cervical and lumbar regions [16].

### 2.2. Literature Search

A literature search was conducted in DBpia for Korean articles and EMBASE, PubMed, and Science Direct for studies published abroad to identify academic articles (excluding degree dissertations). The search keywords were a combination of “spinal cord injury (SCI)”, “pain”, “exercise”, “disability”, “paraplegia”, and “tetraplegia”. In the Science Direct database, the search was conducted using the Advanced Search feature, including all keywords under “Find articles with these terms” and combining keywords in “Title, abstract or author-specified keywords”.

The search was conducted from 18–21 September 2023. There were no restrictions on the publication year for Korean and non-Korean literature, but the language for non-Korean articles was limited to English. Out of 191 articles found, 54 were Korean, and 137 were non-Korean. Additionally, five studies in the journal “Spinal Cord,” [17] published by the International Spinal Cord Society, were included as “Additional records identified through other sources” in the non-Korean literature search. The literature search was carried out independently by one researcher and then reviewed by a second researcher.

### 2.3. Literature Selection and Categorization

**Figure 1:**
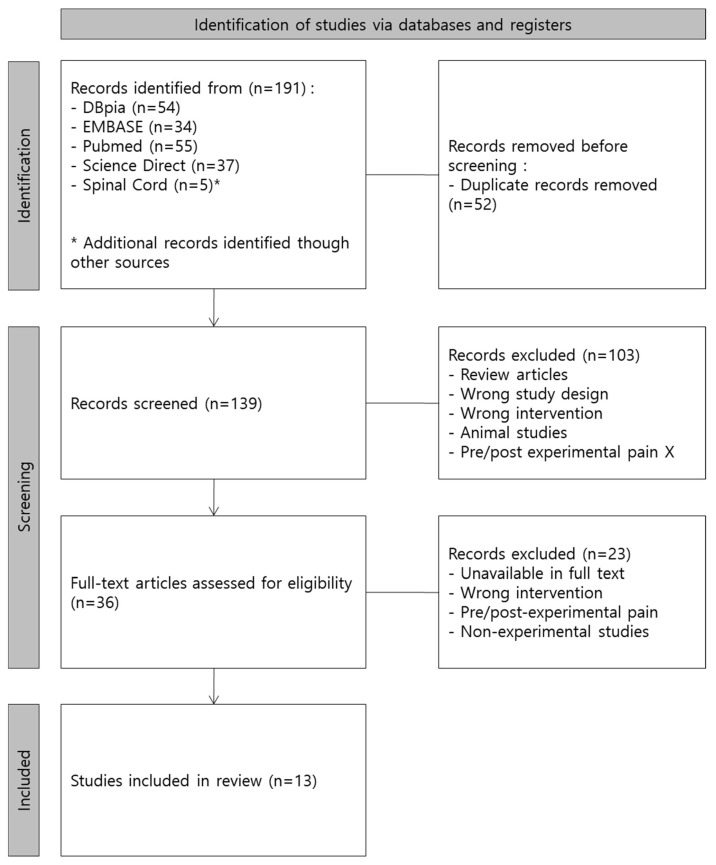
PRISMA 2020 flow diagram for new systematic reviews, which included searches of databases and registers only.

The selection and classification of literature were conducted according to the PRISMA 2020 flow diagram for new systematic reviews, which included searches of databases and registers only (Figure 1 and Table 2). In the identification step, 52 duplicate search results were excluded, and 139 studies remained. In the screening step, the titles and abstracts of the studies were reviewed to determine eligibility per the PICOS, and the full texts of the studies deemed eligible were reviewed. The exclusion criteria in the second screening step were as follows: (1) literature reviews and meta-analysis studies, (2) animal studies, (3) studies with full text unavailable, (4) non-experimental studies, (5) inappropriate or unclear pain criteria for participants, (6) interventions not involving exercise, and (7) no or unverifiable outcomes. No automation tools were used in the selection process. The classification was carried out independently by one researcher and then comprehensively reviewed by a second researcher for appropriateness and completeness. Through this process, 13 studies were selected, including 1 Korean and 12 non-Korean articles.

### 2.4. Data Extraction

The full texts of the 13 selected studies were reviewed, and data were extracted per the PICOS strategy. The extracted data were organized according to first author (year), study design, study population, number of participants (N), intervention details, duration of intervention, assessment items and instruments, and outcomes. In evaluating the pain characteristics within the study populations, a thorough examination was conducted to ascertain whether the criteria included or excluded items specifically related to upper body pain. In cases where this information was absent or unclear, a meticulous review was undertaken to identify whether the study provided data on pain measured before the intervention. This analysis encompassed both the “study parameters (instrument)” and “results” sections. Two studies reported self-reported pain for only a subset of the participants.

In our systematic review, we extracted data using a standardized approach, focusing on the key aspects defined by the PICOS framework. Each selected study was analyzed for its methodological details, including study design, participant demographics, interventions, outcomes, and, crucially, the instruments used for measuring outcomes. Below, we summarize the commonly used research instruments in the included studies, emphasizing their purpose and significance in the context of spinal cord injury and pain assessment.

- Wheelchair User Shoulder Pain Index (WUSPI): Used in seven studies, the WUSPI is a self-reported measure designed to assess shoulder pain severity and its impact on the daily activities of wheelchair users. This tool includes questions on pain intensity during various movements and is highly relevant for tracking the pain dynamics in individuals with SCI.
- Performance-corrected WUSPI (PC-WUSPI): A variation of the original WUSPI, the PC-WUSPI adjusts for participants’ performance capability, providing a more tailored assessment of pain related to shoulder use. Four studies utilized it.
- 36-Item Short Form Survey (SF-36): This broad health survey was used in three studies to measure aspects of quality of life related to physical and mental health. In the context of SCI, the SF-36 helps delineate how pain affects general well-being.
- Visual Analog Scale (VAS): Used alongside WUSPI in two studies, the VAS is a straightforward measure in which participants rate their pain intensity on a scale, typically from ‘no pain’ to ‘worst imaginable pain.’ It is a widely accepted instrument for its simplicity and effectiveness in pain assessment.

## 3. Results

Our systematic review analyzed 13 articles that collectively studied a diverse population of spinal cord injury (SCI) patients. The most common type of study design among the included studies was a randomized controlled trial (six studies, 46%), followed by pre–post comparative experiments (five studies, 38%), a follow-up study for a randomized controlled trial [12] (one study, 8%), and a time-series design (one study, 8%).

The duration of SCI, spinal cord lesion site, complete/incomplete injury, and American Spinal Injury Association Impairment Scale grades varied depending on the inclusion criteria. Six studies (46%) had upper body pain-related criteria in the inclusion criteria, and two of these studies selected participants who had positive results on the functional shoulder impingement syndrome test. Functional shoulder impingement syndrome is one of the most common shoulder disorders among patients with SCI who use a wheelchair. It appears to have been employed as an inclusion criterion due to the persistent strain on the shoulder joint, leading to overuse of surrounding muscles and consequent muscle fatigue. This chronic strain exacerbates the condition, culminating in the development of shoulder impingement syndrome [29]. The pain-related criteria in the included studies were as follows:

1. Positive results on shoulder impingement tests such as Neer’s impingement test, Hawkins–Kennedy test, and empty can test;
2. Impact on one or more functional tasks due to unilateral or bilateral shoulder pain;
3. Moderate shoulder or upper limb pain for a specified period.

The point of pain common in all three criteria was the “shoulder”; this may be attributed to the previous findings that more than half of the patients with SCI suffer from shoulder pain [2,4,30]. The type of pain was generally described as “shoulder pain”, and one study distinguished between neuropathic pain and nociceptive pain.

The duration of intervention ranged from 3 weeks to 9 months, and many studies generally used a relatively long-term intervention, with nine studies (69%) using an intervention lasting 10 weeks or longer. The most common intervention frequency was three times weekly (nine studies, 69%). The contents of the intervention were classified as follows: (1) ergometer, (2) strengthening exercise (bodyweight exercises, resistance exercises), and (3) hybrid exercise (upper limb + lower limb, ergometer + resistance training). Tools used during the experiments were an ergometer (six studies), Theraband (four studies), and hand weights (two studies). Hand weights were used in the same experiments as Theraband. One study used a treadmill, functional electrical stimulation leg cycle, and bodyweight exercise. In studies that did not directly mention the tool used, resistance training was used as the intervention; therefore, the tools used can be inferred based on the exercise motions. Eight studies used muscle training as the intervention, and six specified the exact targeted areas for exercise or motions. Table 3 shows the studies’ targeted areas and motions.

Changes in pain were measured using the Wheelchair User Shoulder Pain Index (WUSPI) (seven studies), performance corrected-WUSPI (four studies), and 36-item short form survey (three studies), and a visual analog scale was used along with WUSPI in two studies [31,32]. The most frequently used parameter, along with pain, was quality of life (Health-Related Quality of Life). Eight types of instruments were used in eight studies, which was justifiable considering that findings suggested a negative correlation of WUSPI with physical and psychological health and overall quality of life and satisfaction, and viewed pain as a factor adversely affecting the quality of life in patients with SCI [33]. Other study parameters included physical markers during exercise (e.g., heart rate, peak oxygen uptake, %peak watts), muscle activity, strength, muscle endurance, upper extremity functionality, fatigue, self-efficacy, physical activity, level of participation, stress, depressive mood, pain interference, and body composition.

## 4. Discussion

This systematic review aimed to assess the effectiveness of exercise interventions in alleviating upper body pain among spinal cord injury (SCI) patients. The results from the review of 13 articles involving a diverse population of SCI patients provide substantial evidence supporting the efficacy of these interventions. Specifically, the studies demonstrated that moderate to high-intensity resistance exercises are particularly beneficial in reducing pain levels in the upper body, which includes critical areas such as the shoulders and upper limbs. The findings highlight the potential for structured exercise programs to be incorporated as a key component of pain management strategies for SCI patients. These interventions not only assist in pain reduction but also contribute to improving overall physical function and quality of life. The beneficial effects of exercise underscore its importance as a non-pharmacological treatment option that can be tailored to individual patient needs based on the severity and type of injury. The results of our systematic review following a certain set of criteria and processes show that exercise interventions effectively reduced pain in patients with SCI with upper body pain, and exercise was identified as essential for patients with SCI and shoulder pain. Most studies included strength training, supporting the effectiveness of incorporating it into exercise programs for patients with SCI, irrespective of injury location or severity. Moderate to high-intensity resistance exercises targeting specific muscle groups enhance muscle strength [34,35]. In some studies that followed pain in patients post-intervention, either no immediate or follow-up changes in pain or an increase in pain was observed during the follow-up period, and this appears to be associated with a decline in exercise participation after the intervention period [36], highlighting the importance of strategies to motivate continual exercise participation and habit formation.

Changes in pain were adopted and measured as secondary outcomes in most studies due to the subjective nature of pain, which presents challenges in quantitatively and objectively measuring it in experimental settings. Hence, researchers presumably aimed to demonstrate the relationship and validity by measuring and analyzing pain alongside outcomes that provide numerical values, such as muscle strength or activity. Most instruments used for pain assessment are self-reported scales, and assessment generally relies on the statements of study participants. Consequently, using pain as a primary outcome might lead to concerns about the accuracy and reliability of the results. Therefore, there is a need for the development of validated pain instruments that can measure changes in pain as a primary outcome, and experimental studies using pain as the primary outcome should be conducted to prevent and alleviate pain in patients with SCI.

Thus, we sought to examine musculoskeletal pain in the upper body in patients with SCI by analyzing existing studies. However, neuropathic pain and other pain types were included in our analysis due to the difficulty of determining whether shoulder pain in patients with SCI is musculoskeletal pain [16] and the abundance of studies that do not specify the type of pain. Musculoskeletal pain is the most common cause of joint pain, and the cause of chronic pain must be identified to provide appropriate treatment [37]. Thus, we recommend that future studies use the Leeds Assessment of Neuropathic Symptoms and Signs to enhance the reliability of experimental studies. The Leeds Assessment of Neuropathic Symptoms and Signs allows the researcher to distinguish between neuropathic pain and nociceptive pain [38], and it can be used to assess whether shoulder pain in patients with SCI is neuropathic pain or nociceptive musculoskeletal pain [39]. Using this instrument to accurately distinguish the cause of shoulder and upper body pain when enrolling study participants would enable researchers to produce more accurate results regarding the cause of pain.

Furthermore, while we limited the point of pain to the upper body, the site of pain was limited to the shoulder due to the participant inclusion criteria that reflected that shoulder pain is the most common pain experienced by patients with SCI [1,4]. The shoulder muscles were also the primary targets for muscle-strengthening exercises. However, given that upper body pain experienced by patients with SCI may occur in various places, including the shoulders, wrist, hands, and elbows, and injury in one area influences other areas [7], increasing overall upper body strength and preventing pain is essential. Future experimental studies should be conducted to evaluate and strengthen the muscles of the entire upper body, including the back, abdomen, and chest, of patients with SCI, and appropriate exercise interventions should be applied. Nevertheless, the increased load due to propelling wheelchairs in environments with high speeds and steep inclines leads to an increase in the range of motion and muscle activity of the trunk flexion and extension rather than the range of motion of the upper limb joints, and core muscles need to be mobilized to prevent injury [7,31,40], which is consistent with previous findings that emphasize the need to strengthen the abdominal muscles and spinal extensors for this purpose [41].

The WUSPI designed by Lee et al. [42] is used to assess shoulder pain in wheelchair users and was the most frequently used instrument in the included studies. Curtis et al. [43] adapted the WUSPI into Korean, using terminology that reflects Korea’s cultural characteristics while maintaining the original’s integrity, and validated the instrument for measuring shoulder pain in wheelchair users in Korea. Both the original WUSPI and the Korean version consist of 15 items, categorized into mobility-related items such as “moving from bed to wheelchair” and “pushing a wheelchair for more than 10 min” and daily activity-related items such as “putting on pants” and “washing the back”. According to Park and Cho [44], 570,462 people with physical disabilities, including those with SCI, are economically active (48.0%), with an employment rate of 46.2%. Despite the active participation of almost half of these individuals in social and economic activities, the WUSPI only contains one item for this: “activities at work/school”; this indicates a gap in identifying and measuring the pain wheelchair users might experience in social and economic contexts. Furthermore, living standards have changed significantly since the time of development of the WUSPI, as the penetration rate of cellphones/smartphones among people with physical disabilities has reached 95.2% [45], calling for new items that reflect such shifts. Therefore, new items should be developed, or existing items should be modified in this context.

According to Lee and Shin [14], patients with SCI who use a wheelchair have significantly stronger shoulder muscles, especially shoulder external rotation, and right internal and external rotation, compared to those capable of incomplete ambulation. These results are inconsistent with previous findings that the use of a wheelchair induces continuous stress on the shoulder and relevant disorders and adversely affects the surrounding muscles. The reason was attributed to the fact that the study population consisted of patients with SCI without shoulder injury or pain. That is, the use of a wheelchair by users without a history of shoulder injury seems to have had positive effects on shoulder rotation and strength. Conversely, inappropriate use of manual wheelchairs may induce pain, and it is important to choose a manual wheelchair suitable for the user’s body shape and conditions.

A user survey was conducted on the perceived need and satisfaction with manual wheelchairs among three patients with varying degrees of SCI who are representative of the SCI population [43]. In addition, a study in Korea proposed that the standard wheelchair design is ergonomically appropriate for the body figures of Koreans by measuring the specifications of wheelchairs marketed in Korea and comparing them with anthropometric measurements [44]. However, these were standard specifications obtained from patients with temporary disabilities and may be inappropriate for patients with SCI who must use wheelchairs for prolonged periods and durations. Lee and Yoo [45] recommend the use of lightweight, ergonomically suitable manual wheelchairs to prevent injuries and disorders in individuals with SCI who are at risk of shoulder pain, as well as power assist devices designed to avoid deformities in the upper limbs and enhance mobility. Therefore, it is essential to develop guidelines that enable individuals with SCI to self-assess their physical condition, presence and degree of shoulder pain, and muscle strength. Additionally, it is essential to establish an environment where they can choose manual wheelchairs with ergonomic structures and specifications suited to their assessment results. In particular, there is a call for developing new manual wheelchairs equipped with features that can prevent and alleviate shoulder pain. Such advancements in assistive technology are crucial for improving the quality of life for SCI patients, emphasizing the practical implications of our findings.

While the study is significant for examining correlations among exercise interventions, muscle strength improvements, and pain relief, it is important to acknowledge that researchers’ subjective biases in the selection and extraction of study data could not be completely eliminated. This acknowledgment underscores the need for cautious interpretation of the results.

## 5. Conclusions

Our study underscores the significant impact of exercise interventions in alleviating upper body and shoulder pain among spinal cord injury (SCI) patients. Exercise interventions, particularly moderate- to high-intensity resistance exercises, play a crucial role in managing pain for individuals with spinal cord injuries. However, the sustained effectiveness of these interventions is often compromised by a decline in exercise adherence after the conclusion of structured programs. This underscores the necessity of developing innovative strategies that maintain exercise engagement and promote the formation of long-term habits for ongoing pain management.

The inherent challenges in pain measurement, owing to its subjective nature, call for the development of validated instruments capable of quantitatively assessing pain as a primary outcome. This advancement is crucial for refining research focused on the effective prevention and management of pain in SCI populations. Moreover, our findings reveal the necessity of distinguishing between neuropathic and nociceptive musculoskeletal pain to tailor treatment approaches more precisely.

Given the prevalence of shoulder pain in SCI patients, it is essential to adopt a comprehensive approach that considers the entire upper body. Such an approach could potentially offer more holistic pain management solutions, addressing the multifaceted nature of musculoskeletal pain. However, while the review underscores the importance of ergonomic considerations in manual wheelchair design, improving these designs, although beneficial, was not a primary focus of this review. Thus, the suggestion of enhancing wheelchair ergonomics, while valid, does not stem directly from the findings of this review but rather is a recognized need in the field.

Furthermore, the review highlights certain limitations in current pain assessment tools used in SCI research. Addressing these limitations could contribute to better clinical practices and research outcomes. However, it is important to clarify that enhancing these tools alone is unlikely to directly improve the overall quality of life for SCI individuals, as suggested earlier. Such improvements must be part of broader, multi-faceted interventions that include but are not limited to better pain management strategies.

Lastly, the review did not conclusively differentiate between neuropathic and nociceptive musculoskeletal pain, although it does suggest that understanding these differences could be crucial for tailoring pain management strategies. It is crucial to reiterate that the primary conclusion of this review focuses on the effectiveness of exercise interventions in managing pain, rather than on the distinctions between types of pain.

## Figures and Tables

**Table 1:** Intervention protocol.

P	Participants	Patients with SCI with Upper Body Pain
I	Intervention	Exercise
C	Comparison	Control group
O	Outcome	Changes in pain
S	Study design	Experimental study

**Table 2:** Effectiveness of exercise programs for alleviation of upper body pain in patients with SCI.

First Author (Year)	Study Design	Study Population	N	Intervention Details	Duration of Intervention	Study Parameters (Instruments)	Outcomes
Kim, S.C. et al. (2018) [18]	Pre–post comparative experiment	- Duration of SCI ≥ 1 year - Complete/incomplete injury between C8–T12 - Minimum two positive results in functional impingement syndrome test - Cervical (*n* = 3), thoracic (*n* = 6), lumbar (*n* = 1) - AIS-A (*n* = 9), - AIS-C (*n* = 1)	10	- Exercise to decrease upper trapezius activity and enhance serratus anterior activity (1 type) - Exercises to correct imbalance between trapezius muscles (4 types) - 1 set = 10 reps, total 3 sets, 30 s rest between sets	10 weeks, 3 times a week	(1) Shoulder pain (WUSPI) (2) Trapezius and serratus anterior activities (wireless surface ECG)	(1) Significant reduction in shoulder pain (WUSPI) *p* = 0.02 (<0.05) 20.9 ± 10.9 → 15.4 ± 10.46
Nightingale, T.E. et al. (2018) [19]	RCT	- Inactive (PAL < 1.60) chronic SCI participants over 1 year - SCI T2–T4	21	Experimental (*n* = 13): - Moderate-intensity upper body exercise using a portable desk-arm crank ergometer (home training) - 45 min, adjustable intensity range (~60% Vo2peak for 3 weeks → ~65% Vo2peak for 3 weeks) Control (*n* = 8): - Maintain daily life	6 weeks, 4 times a week	(1) HRQOL (SF-36) - PCS - MCS - QALY (2) Shoulder pain (PC-WUSPI) (3) Fatigue (FSS) (4) Exercise Self-efficacy (ESES)	(2) Greater change in shoulder pain (PC-WUSPI) score in the experimental group - Experimental group: 19 ± 21 → 14 ± 15 △ (90%CI) = −5 (−16.6) - Control group: 13 ± 11 → 13 ± 13 △ (90%CI) = 0 (−4.4) (△: change scores)
Gee, C.M. et al. (2022) [20]	Multi-center RCT	- Traumatic SCI with complete motor impairment - C4–T6 - Greater carotid–femoral pulse wave velocity than middle norms of non-disabled counterparts of the same age group	28	Active upper limb exercise (*n* = 14): - Arm-cycle ergometry (ACET) Passive lower limb exercise (*n* = 14): - Body weight supported treadmill training (BWSTT)	72 sessions, 3–5 weeks	(1) QoL (LiSAT-9, SWLS) (2) LTPA (MVPA of LTPAQ-SCI) (3) Pain (SF-36) (4) Affect (PANAS-positive/negative) (5) Self-efficacy (questionnaire) (6) Independence (SCIM-III) (7) Participation and Autonomy (IPAQ)	(3) Pain (SF-36) - Significant reduction in pain in ACET compared to BWSTT *p* = 0.022 - Significant reduction in pain in ACET from baseline after 72 sessions *p* = 0.009 - No significant difference between after 72 sessions and follow-up in both interventions - Upper limb exercise (ACET): 39.71 → 45.36 → 47.60 - Lower limb exercise (BWSTT): 58.07 → 50.68
Van Straaten, M.G. et al. (2014) [21]	Pre–post comparative clinical trial	- Manual wheelchair users with shoulder pain (mean 9 years) - C6-7 (*n* = 1), T2-7 (*n* = 5), below T8 (*n* = 9), post-polio syndrome (*n* = 1) - Used manual wheelchair as primary means of mobility for ≥1 year - Shoulder pain identified as a major cause of functional impingement syndrome (positive result on relevant test)	16	- Resistance band (Theraband) exercises for muscle strengthening - Focused on serratus anterior, scapular retractor depressor muscles, and glenohumeral external rotator - 1 set = 30 reps, total 3 sets, 30 s rest between sets	12 weeks, 3 times a week	(1) Shoulder pain (WUSPI) (2) Upper extremity function (DASH, SRQ)	(1) Shoulder pain (WUSPI) - Significant reduction between baseline and 12 weeks - Significant reduction between baseline and 24 weeks - No significant change between 12 weeks and 24 weeks 22.8 → 12.5 → 10.9
Mulroy, S.J. et al. (2011) [22]	RCT	- SCI ≥ 5 years - Impact on one or more tasks due to unilateral or bilateral shoulder pain (e.g., mobility, use of manual wheelchair) - Manual wheelchair used at least 50% of time for movement	58	Experimental (*n* = 26): - Shoulder exercise program for optimization of exercise and daily motions (home training) - Stretching, warm-up, resistant shoulder exercise (hypertrophy, endurance) - Provide advice (method) on optimal daily life movements Control (*n* = 32): - Watch 1-h video on shoulder anatomy, injury mechanisms, and shoulder pain management	12 weeks, 3 times a week	(1) Shoulder pain (WUSPI, VAS) (2) Muscle strength (shoulder torque) (3) Activity (PASIPD, wheelchair propulsion speed) (4) QoL (SII, SQOL, SF-36)	(1-1) Shoulder pain (WUSPI) - Reduction to ⅓ of baseline immediately after intervention in the experimental group - No change between immediately after intervention and baseline in the control group - Reduced pain at 4 weeks after intervention than immediately after intervention in the experimental group - No change between 4 weeks after intervention and immediately after intervention in the control group (1-2) Shoulder pain (VAS) - Reduction to ⅓ of baseline immediately after intervention in the experimental group - No change between immediately after intervention and baseline in the control group
Vestergaard, M. et al. (2022) [23]	One-group pre–post comparative trial (Validation)	- SCI with paraplegia - Complete/incomplete SCI - Traumatic/non-traumatic SCI - WUSPI ≤ 45	7	- Hybrid high-intensity interval training - FES leg cycle + arm ski ergometer - 4 sessions × 4 min, 2-min active rest between sessions (1) Leg exercise (2) arm exercise (3) Leg + arm exercise (hybrid)	8 weeks, 3 times a week	(1) AE (2) Participant acceptability (PACES) (3) Shoulder pain (PC-WUSPI) (4) Shoulder/arm/hand pain (NRS) (5) Training intensity (% peak watts) (6) Attendance (7) Vo2peak (8) Physical activity (SCI-LTPAQ) (9) HRQOL (SF-36) (10) Fatigue (MFI-20)	(3) Shoulder pain (PC-WUSPI) - Slight increase in average PC-WUSPI △(%) = 9 - One participant showed a large increase in PC-WUSPI after intervention △(%) = 204
Hicks, A.L. et al. (2003) [12]	RCT	- Traumatic SCI - C4~L1 - ASIA A-D - Paraplegia or quadriplegia	23	Experimental (*n* = 11): (1) arm ergometer exercise - 2 bouts, BRG 3–4, 15–30 min per bout (2) Resistance training - 3 sets, 70–80% of maximum weight Control (*n* = 12): - Education session	9 months, 2 times a week	(1) Arm ergometry performance (2) Muscle strength (max load could be lifted) (3) Stress (PSS) (4) Depression (CES-D) (5) Physical self-concept (questionnaire) (6) Pain (SF-36) (7) Perceived health (SF-36) (8) QoL (PQOL)	(6) Pain (SF-36) - Experimental group showing lower pain levels versus control group after intervention *p* < 0.01 - Experimental group △(%) = −9.5 ± 18.79 - Control group △(%) = 12.8 ± 17.96
Ditor, D. S. et al. (2003) [24]	RCT Follow-up	- Chronic SCI (3~23 years) - C5~T12 - ASIA A-D - Completion of previous study spanning 9 months (AL Hicks, 2003)	7	Same intervention used in a previous study (AL Hicks, 2003) [12]	3 months, 2 times a week	(1) Exercise adherence (sessions attended) (2) QoL (PQOL) (3) Pain (SF-36) (4) Stress (PSS)	(3) Pain (SF-36) - Generally increased pain between completion of previous study and follow-up (3 months) *p* = 0.07 - Significant reduction in session participation between completion of previous study and follow-up (3 months) *p* < 0.01 - Significant negative correlation between pain score at completion of previous study and follow-up (3 months) *p* < 0.01
Cardenas, D.D. et al. (2020) [25]	Single-blinded RCT	- SCI ≥ 1 year - Use of manual/powered wheelchair for at least 50% of time when moving - Shoulder pain at least “moderate” (score ≥ 4, 0–10 NRS) in the past 3 months	25	Experimental (*n* = 13): - stretching + muscle strengthening exercise - Resistance band, hand weights Control (*n* = 12): - Watch 1 h video on shoulder anatomy, injury mechanisms, and shoulder pain management - Provide relevant printouts	12 weeks, 3 times a week	(1) Interview about shoulder pain (NRS) (2) Shoulder pathology (PESS-dominant/non-dominant) (3) Functional limitations of upper limbs (DASH) (4) The effect of pain on ADLs (MPI-Interference) (5) Shoulder pain (WUSPI) (6) Well-being (PHQ-9) (7) Depressive symptoms (BDI) (8) Patient Global Impression of Change Scale	(5) Shoulder pain (WUSPI) - Generally decreasing pain in the experimental group - Experimental group: 68.76 → 53.60 → 48.26 - Control group: 52.84 → 39.23 → 52.13
Kemp, B.J. et al. (2011) [3]	RCT	- SCI ≥ 5 years - Shoulder pain ≥ 5 years on average - Paraplegia - Impact on one or more functional tasks due to unilateral or bilateral shoulder pain	58	Experimental (*n* = 26): - Home training consisting of 3 types of stretching and 4 types of strengthening exercise - Elastic band, hand weights - Wheelchair-bound exercise - Provide a list of various activities aiming towards reducing shoulder joint stress Control (*n* = 32): - Provide information about shoulder joint and maintaining function through videos and printouts	12 weeks, 3 times a week	(1) Shoulder pain (WUSPI) (2) Activities (SII) (3) QoL (Likert-type scale)	(1) Shoulder pain (WUSPI) - No significant change in control group - Significant change in the experimental group *p* < 0.001 - Significant interaction between WUSPI change and SII change - Significant interaction between WUSPI change and QoL change
Nash, M.S. et al. (2007) [26]	Pre–post comparative time-series	- Male - Complete motor impairment, paraplegia - T5–T12 - AIS A-B - Use of wheelchair for mobility - Mild to moderate upper limb pain during daily life	7	Circuit resistance training	16 weeks, 3 times a week	(1) Upper limb endurance (Vo2peak by ergometer) (2) Anaerobic power (peak/mean power by ergometer) (3) Upper-extremity dynamic strength (1-RM) (4) pain (WUSPI)	(4) Shoulder pain (WUSPI) - Significant reduction in shoulder pain *p* = 0.008 31.8 ± 23.5 → 5.0 ± 7.7 - Some (N = 3) reported pain to be (almost) completely resolved after intervention
Norrbrink, C. et al. (2012) [27]	Pre–post comparative experiment	- Wheelchair user with thoracic or lumbar injury - Paraplegia ≥ 2 years - T5–L1 - Inclusion of pain protocol (*n* = 8) - Those not included but have intermittent musculoskeletal pain (*n* = 3)	13 (8)	- Double falling ergometer exercise tailored to lower limb motor impairment - Up to 50 min for each session - Warm-up, 4 sessions × 6–7 min (interval), cool-down. - 70–100% of HR	10 weeks, 3 times a week	(1) - peak heart rate - distance - average intensity (2) classifying and assessing pain (ISCIPDS:B) - neuropathic - nociceptive musculoskeletal (3) Shoulder pain (WUSPI) (4) Global pain-relieving effect (PGIC) (5) Pain interference (ISCOS Basic pain data set) (6) QoL (ISCOS quality of life basic data set ver1.0)	(2-1) Neuropathic pain - N = 7 - Reduction in average pain intensity 5 → 3 - No change in number of days in pain - Reduced number of pain sites (2-2) Nociceptive musculoskeletal pain - N = 5 - Reduction in average pain intensity 4 → 0 - Reduction in number of days in pain 3) Shoulder pain (WUSPI) - N = 5 - Reduction in average WUSPI score 37 → 18
Serra-Añó, P. et al. (2012) [28]	Time series design	- Chronic thoracic SCI - Complete lower limb motor impairment - AIS A-B - Full-time manual wheelchair user - Participants who reported shoulder pain at baseline (WUSPI, *n* = 8)	15 (8)	Time series design: Measurement 1 → Control (8 weeks) → Measurement 2 → Experiment (8 weeks) → Measurement 3 Experimental: - Resistance training - Including warm-up and cool-down - 3 sets, 8–12 reps per set - 8 motions Control: - Not following training protocol.	8 weeks each, 3 times a week	(1) Isometric and isokinetic shoulder muscle strength (2) Body composition - arm fat-free mass - arm fat mass (3) Shoulder pain (PC-WUSPI) (4) Upper-limb functionality (DASH)	(3) Shoulder pain (PC-WUSPI) - Significant reduction after intervention (measurement 3) *p* < 0.05

Abbreviations: AIS, American Spinal Injury Association Impairment Scale; WUSPI, Wheelchair User Shoulder Pain Index; HRQOL, Health-Related Quality Of Life; SF-36, 36-Item Short Form Survey; PCS, Physical Component Summary; MCS, Mental Component Summary; QALY, Quality Adjusted Life Years; PC-WUSPI, Performance-corrected WUSPI; FSS, Fatigue Severity Scale; ESES, Exercise Self-Efficacy Scale; LiSAT-9, Life Satisfaction Questionnaire 9; SWLS, Satisfaction With Life Scale; LTPA, Leisure Time Physical Activity; MVPA, Moderate-Vigorous intensity Physical Activity; PANAS, Positive and Negative Affect Schedule; SCIM, Spinal Cord Independence Measure; IPAQ, Impact on Participation and Autonomy Questionnaire; DASH, Disabilities of the Arm, Shoulder, and Hand Index; SRQ, Shoulder Rating Questionnaire; VAS, Visual Analog Scale; PASIPD, Physical Activity Scale for Individuals with Physical Disabilities; SII, Social Interaction Inventory; SQOL, Subjective Quality of Life Scale; AE, Adverse Events; PACES, Physical Activity Enjoyment Scale; NRS, Numeral Rating Scale; SCI-LTPAQ, SCI Leisure Time Physical Activity Questionnaire; MFI-20, Multi-Dimensional Fatigue Inventory; PSS, Perceived Stress Scale; CES-D, Centre for Epidemiological Studies Depression Scale; PQOL, Perceived Quality Of Life scale; PESS, Physical Examination of the Shoulder Scale; ADLs, Activities of Daily Living; MPI-Interference, interference subscale of the Multidimensional Pain Inventory; PHQ-9, Patient Health Questionnaire 9; BDI, Beck Depression Inventory; Vo2peak, peak oxygen uptake; 1-RM, one-repetition maximum; ISCIPDS:B, International SCI Basic Pain Data set; PGIC, Patient Global Impression of Change scale; ISCOS, International Spinal Cord Society.

**Table 3:** Targeted areas of exercise and motions.

First Author (Year)	Type of Exercise	Targeted Area	Exercise Motions
Kim, S.C. et al. (2018) [18]	Bodyweight exercise	- Upper trapezius - Serratus anterior	- Shoulder flexion in a side-lying position - Shoulder external rotation in a side-lying position - Horizontal abduction in a side-lying position and shoulder external rotation - Shoulder extension in a prone position - Shoulder retraction in a quadruped position
Van Straaten, M.G. et al. (2014) [21]	Resistance exercise	- Serratus anterior - Scapular retractors and depressors - Glenohumeral external rotator	
Mulroy, S. J. et al. (2011) [22]	Resistance exercise		Hypertrophy exercises - Shoulder abduction - Shoulder external rotation - Endurance exercises - Shoulder elevation in the scapular plane - Scapular retraction
Hicks, A.L. et al. (2003) [12] Ditor, D.S. et al. (2003) [24]	Resistance exercise	Forearm/wrist, biceps, back, chest, abdominal, shoulder, triceps, and leg (only when suitable)	- Wall pulley exercises - Free weights - Equalizer weight machine
Nash, M.S. et al. (2007) [26]	Resistance exercise		- Overhead press - Horizontal row - Horizontal butterfly - Biceps curl - Latissimus pull-down - Triceps press
Serra-Añó, P. et al. (2012) [28]	Resistance exercise	Rotator cuff	- Lateral raise - Latissimus pull down - Horizontal row - Biceps curl - Internal and external rotation with 90° of abduction - Internal and external rotation in the neutral position

## Data Availability

The data that support the findings of this study are available from the corresponding author (D.K.) upon reasonable request.
